# Spectrum of parathyroid disorders in Pakistan: a review article

**DOI:** 10.1097/MS9.0000000000003007

**Published:** 2025-02-07

**Authors:** Umaima Wasim, Maria Waseem, Usha Kumari, Varsha Kumari, Abdul Moiz Sahito, Shanzay Zahid, Ifrah Afzal, Somina Shaikh, Shaheer Alam, FNU Danish, Aarash Khan

**Affiliations:** a Ziauddin University, Karachi, Pakistan; b Dow University of Health Sciences, Karachi, Pakistan; c Nowshera Medical College, Nowshera, Pakistan; d Jinnah Sindh Medical University, Karachi, Pakistan; e Herat University, Herat, Afghanistan

**Keywords:** hyperparathyroidism, hypoparathyroidism, parathyroidectomy, parathyroid hormone

## Abstract

Parathyroid abnormalities and gland diseases are classified as hyperparathyroidism and hypoparathyroidism. These conditions are often discovered incidentally during screenings for abnormal serum calcium levels, typically conducted for renal or bone diseases and other disorders. Parathyroid hormone (PTH) plays a crucial biological role in maintaining ionized calcium and phosphate levels within the normal range by activating specific receptor-mediated cell responses throughout the body. Compared to the Western world, Pakistan has significantly fewer publications on parathyroid diseases. Most available research comprises case reports, small case series, or retrospective studies, often including approximately 100 studies. In this article, we describe the demographics and varied clinical presentations of parathyroid gland disorders in the Pakistani population. Additionally, we provide a summary of biochemical parameters and localization strategies used in these cases. A compiled record of parathyroid gland disorders in Pakistan is currently lacking, and this article aims to bridge that gap by presenting a comprehensive review of all reported cases. This review includes a summary of 54 studies, comprising retrospective studies and case reports, highlighting the clinical variability of parathyroid (PTH) disorders in Pakistan. According to the literature, 0.5% of patients were asymptomatic, while the mean duration of symptoms among symptomatic patients was 3.03 years. Unlike Western populations, laboratory findings from Pakistani patients show elevated levels of serum calcium, serum PTH, and serum alkaline phosphate. Common clinical features included abdominal pain, dysphagia, and vitamin D deficiency, along with constitutional symptoms such as arthralgias, myalgias, fatigue, and headache. Psychiatric complications, particularly clinical depression, and pathological fractures emerged as the most frequent complications associated with PTH disorders in Pakistan.

## Introduction

Essential minerals such as calcium and phosphorus are primarily regulated hormonally through intestinal absorption, renal resorption, and bone turnover. These processes are influenced by interactions between hormones and receptors, including parathyroid hormone (PTH), PTH-related peptide, 1,25-dihydroxyvitamin D, calcitonin, fibroblast growth factor 23 (FGF23), blood calcium levels, and calcium-sensing receptors (CaSR)^[1]^. PTH regulates ionized calcium and phosphate levels by activating receptor-mediated responses in a range of cells, including mesenchymal stem cells, osteoblasts, T-cells, and osteocytes^[2]^. It is secreted by the parathyroid glands in response to low calcium levels and is regulated by endocrine feedback. Elevated blood calcium levels activate CaSR, which reduces PTH production, while low calcium triggers the release of PTH^[3]^. PTH promotes renal calcium resorption, inhibits phosphate reabsorption, and activates 1,25-dihydroxyvitamin D to increase intestinal calcium absorption^[4]^. Furthermore, 1,25-dihydroxyvitamin D stimulates FGF23 secretion, which inhibits PTH synthesis via negative feedback^[3]^.

**Highlights**

- Parathyroid disorders are common in Pakistan, but the lack of early screening leads to delayed diagnosis.
- Most patients present with the classic symptoms of “bones, moans, and groans,” while only a small percentage are diagnosed at an asymptomatic stage. This contrasts with developed countries, where early testing allows for the early identification and treatment of the disease.
- Notably, mean levels of serum calcium, phosphorus, and parathyroid hormone are higher in Pakistan compared to other populations worldwide.
- This review highlights the spectrum of parathyroid disorders in Pakistan and underscores the scarcity of literature on the subject.

Disorders of PTH homeostasis range from primary hyperparathyroidism (PHPT) to rarer conditions such as pseudohypoparathyroidism (ps-HypoPT)^[5]^. PHPT is often identified incidentally through elevated blood calcium levels caused by excessive PTH production, which affects bones and kidneys^[6]^. While usually asymptomatic, it may present as bone loss or kidney stones^[7]^. Treatment options include surgery (parathyroidectomy) and medications such as bisphosphonates^[6,8]^. Secondary hyperparathyroidism (SHPT), commonly associated with chronic kidney disease (CKD), develops due to metabolic changes such as vitamin D deficiency and dietary alterations^[9]^. Prolonged SHPT may progress to tertiary hyperparathyroidism (THPT), characterized by autonomous PTH production due to parathyroid hyperplasia^[10]^. The surgical management of SHPT and THPT is not well-defined, but parathyroidectomy has shown positive outcomes^[11,12]^.

Hypoparathyroidism (HypoPT) is typically caused by surgical injury and is characterized by hypocalcemia, low PTH, and reduced 1,25-dihydroxyvitamin D levels^[13]^. Treatment involves calcium and vitamin D supplementation, with recombinant PTH (1–84) emerging as a more effective option. Preventing surgical damage to the parathyroid glands is crucial to avoid additional complications^[13,14]^. Ps-HypoPT is a rare parathyroid disorder caused by end-organ PTH resistance, resulting in hypocalcemia and hyperphosphatemia despite normal parathyroid function. It is often associated with brachydactyly, ectopic ossification, and obesity, and requires genetic confirmation and lifelong multidisciplinary care^[15–17]^.

There are fewer studies on parathyroid disorders in Pakistan compared to developed countries. This review emphasizes the need for further research in this area.

## Methodology

This review compiled and analyzed 54 studies, including 11 retrospective studies, 6 cohort studies, 21 case reports, case series, 8 cross-sectional studies, descriptive studies, and a randomized controlled trial. The studies were conducted in tertiary healthcare centers across Pakistan. Data were collected on demographics, clinical presentations, surgical and postoperative outcomes, and biochemical parameters of parathyroid disorders. A comprehensive literature search was conducted using the PubMed database, with the following keywords: “parathyroid disorders,” “hyperparathyroidism,” “hypoparathyroidism,” and “Pakistan.”

The inclusion criteria were studies on parathyroid disorders limited to the Pakistani population, focusing on hyperparathyroidism and HypoPT. Data were presented using statistical terms such as mean ± SD, medians, and ranges.

This approach allowed for a comprehensive compilation of the available evidence on the clinical and biochemical characteristics of parathyroid disorders in Pakistan, as well as the surgical management and outcomes. The findings are intended to contribute to current knowledge and identify potential areas for future research.

## Our findings of this review

### Demographics

A total of 7542 individuals were included in the study, with 58% being female and 41% male. All age groups were considered, ranging from 18 days to 70 years. The mean age at presentation was 42.64 years, and the mean duration of symptoms was 3.03 years. The study included premenopausal and postmenopausal women, the pediatric population, and community-dwelling residents of Pakistan.

### Clinical presentation

The clinical spectrum of parathyroid disorders in Pakistan varies significantly, with a very low incidence of asymptomatic cases. In the Western world, however, parathyroid disorders have evolved into an often asymptomatic condition, with routine screenings of serum calcium levels being common practice. Clinical manifestations of symptomatic disease include osteoporosis, hypercalciuria with vertebral fractures, and nephrolithiasis. In our study, only 0.5% of individuals were asymptomatic^[18–20]^. Gastrointestinal and musculoskeletal complaints were the most prominent in clinical presentations. Gastrointestinal symptoms included recurrent oral ulcers, dysphagia to solids and liquids, diffuse burning epigastric pain exacerbated by food intake or at night, post-prandial bloating, vomiting, and altered bowel and eating habits^[21–27]^. A significant gastrointestinal complication secondary to hypercalcemia, resulting in acute pancreatitis and acute abdomen with considerable mortality, was also reported^[18]^.

Musculoskeletal complaints included backache, inability to walk, proximal muscle weakness, fractures, dysarthria, arthralgia, myalgia, bone pain, joint hypermobility, osteitis fibrosa cystica, carpopedal spasm, myositis syndrome, and soft tissue swelling^[18,20,24,26,28–31]^. Metastatic calcifications were also noted^[32]^. Several skeletal deformities, such as bilateral valgus deformity of the knees, eversion deformity of the ankle joints, and flat feet, were reported^[33]^. Radiological imaging revealed bone abnormalities, including severe osteopenia, osteoporosis, and overt osteomalacia^[20,23,34]^. Recurrent renal stones were also noted^[20,23,35,36]^.

Additionally, patients presented with neurological abnormalities such as headaches, irritability, generalized tonic-clonic seizures, tingling sensations in the lower limbs, and insomnia^[18,25,37]^. Some patients showed cerebral calcifications on imaging^[30]^. Many patients also reported psychiatric abnormalities, including altered mental status, depression, short temper, dysphoria, and disorientation^[21,22,25,37]^. Our overall analysis shows that abdominal pain and dysphagia were the most common gastrointestinal symptoms seen. Constitutional symptoms such as generalized body aches or bone aches, fatigue, and headaches had a higher incidence among patients. A few of the patients reportedly had fractures and were unable to walk.

### Parathyroid disorders in Pakistan: biochemical insights, clinical manifestations, and complications

In Pakistan, biochemical guidelines for diagnosing parathyroid diseases include blood calcium, PTH, alkaline phosphatase (ALP), phosphorus, and vitamin D levels. PHPT is characterized by hypercalcemia and increased PTH, whereas hypocalcemia and low PTH levels signify HypoPT. Elevated ALP suggests greater bone turnover, although phosphorus levels vary by situation. Renal function tests and urine calcium levels aid in identifying secondary causes, such as CKD^[38]^.

Ultrasound (USG) is the first-line imaging modality for localization, with 99 mTc-sestamibi scintigraphy utilized to locate hyperfunctioning glands before surgery. Advanced procedures such as CT, MRI, and 4D-CT are employed for ectopic glands and difficult instances, whereas selective venous sampling is used when other methods fail^[38]^. In Pakistan, cost-effective methods like USG and basic biochemical testing are the most popular, with advanced imaging used by special facilities.

In contrast to Western countries, parathyroid illnesses in Pakistan often become apparent at a later stage due to a lack of awareness and systematic screening. SHPT gets worse by vitamin D insufficiency, which is more common in Pakistan than in Western nations.^[39]^ This delayed detection and lack of early treatments resulted in advanced disease stages, including severe bone abnormalities, fractures, nephrocalcinosis, and kidney stones. Common side effects include osteoporosis, chronic renal disease, depression, neuromuscular difficulties, hypertension, and arrhythmias. These issues emphasize the need for increased awareness, early detection, and prompt treatments in Pakistan. The mean values for various biochemical parameters necessary for diagnosing parathyroid disorders are provided in Table 1.

**Table 1:** Mean value of biochemical parameters on hyperparathyroidism.

Parameter	Mean value	Normal value
Serum calcium (mg/dL)	10.44	8.1–10.4
Serum phosphorus (mg/dL)	7.42	2.5–4.8
Serum parathyroid (pg/mL)	379.83	16–81
Serum alkaline phosphatase (IU/L)	450.25	44–147
Vitamin D 3 (ng/mL)	27.82	Sufficient >30

### Surgical approach

A more recent study done in a tertiary care facility in Pakistan in 2020 found that out of 103 individuals diagnosed with PHPT, 76.7% chose surgical therapy, with the remaining one-fourth choosing medicinal treatment^[40]^. In Pakistan, patients were treated conservatively with IV fluids, calcitonin, bisphosphonates, vitamin D and calcium supplementation, furosemide, and anti-epileptics^[24,25,41,42]^. Another research found that some individuals received both oral prednisolone and subcutaneous calcitonin^[38]^. Among surgical procedures, the most utilized targeted approaches to treat PHPT were the removal of a localized gland (parathyroidectomy), bilateral neck exploration, and a focused unilateral approach^[18,21,37,43]^.

One of the retrospective studies reported the use of bilateral neck exploration in 58.3% of the cases and the unilateral approach in 27% of the patients^[43]^. Another study indicated that access was obtained mostly through the neck and least commonly through a median sternotomy^[19]^. However, the use of endoscope-assisted parathyroidectomy (PTX) and limited neck exploration has been found to gain widespread acceptance to prevent excessive tissue dissection^[37]^. Another safe and efficient minimally invasive technique for the removal of ectopic mediastinal parathyroid adenomas is video-assisted thoracoscopic surgery^[23]^, however, randomized clinical trials outside Pakistan have not demonstrated a consistent long-term benefit of PTX over observation on nonclassical manifestations of parathyroid disorders.

### Pathological features

The analysis of the data from four retrospective cohort studies that included a total of 202 patients with hyperparathyroidism revealed the frequency of parathyroid adenoma, hyperplasia, and carcinoma in 77.2%, 7.92%, and 14.8% of cases respectively show a higher incidence of carcinoma in the developing nations^[18,20,21,43]^. Also, an observational study on 25 patients presented with brown tumor and giant cell granuloma of the mandible was observed in 8% and 4% of patients respectively. Some other features that were also commonly observed include renal stone disease which has been known to occur in 12.5%–40.3% of the patients^[18,19,21,40,43]^ while tonic seizures, cerebral calcification, and hypertonia were some of the pathological changes that were seen to be associated with HypoPT^[41]^.

Due to the higher incidence of CKD, SHPT is more prevalent in men and is one of the major factors that contribute to the development of persistent anemia in end-stage renal disease as in ESRD, PTH levels that are elevated cause damage through alterations in the bone mineral composition.

### Postoperative course

There is a significant dearth of studies on the postoperative course and problems seen following surgery in Pakistan. Fatima and colleagues found that hungry bone syndrome occurred in 6.7% of 103 patients having surgery, which is less than expected, presumably due to preoperative care of Vitamin D deficient individuals^[40]^. Transient hypocalcemia was observed in 18.5% of unilateral explorations and 35.7% of bilateral explorations, with chronic illness occurring in 10%–12.6% of occurrences, along with the development of tetany^[20,21,43]^. To manage calcium levels, all patients received oral calcium supplements after surgery, and only four patients required supplementation for more than 6 months^[43]^. Similarly, in other investigations, temporary hypocalcemia was reported in 14.5%–16.8% of people following thyroidectomy, whereas persistent hypocalcemia ranged from 1.8% to 2.9%^[44,45]^. One study similarly found persistent PTH increase postoperatively, but it was normalized in 60% of patients during the first 2 weeks following surgery^[46]^.

## Limitations

Limitations of our literature review involved insufficient data, sparse documentation on the postoperative course, and long-term follow-up. Moreover, statistical data could not be pooled in a major part of the article as there was a lack of homogeneity among studies.

## Conclusion

Based on our research, the mean duration of symptoms in the symptomatic person was 3.03 years while 0.5% of the persons were asymptomatic. Some of the common gastrointestinal and musculoskeletal complications reported include recurrent oral ulcers, dysphagia, epigastric discomfort, bloating, vomiting, backache, inability to walk, proximal muscle weakness, fractures, arthralgia, myalgia, bone pain, and joint hypermobility. The results of laboratory tests show that the levels of calcium, PTH, and alkaline phosphate are high in the blood of Pakistani patients. Psychiatric disorders, including clinical depression and pathological fractures, were identified as the most frequent complications of PTH diseases in Pakistan. About 75% of the patients undergo surgery, which may include a focused unilateral approach, bilateral neck exploration, and parathyroidectomy. Also, endoscopy and video-assisted thoracoscopy are being used with increasing frequency.

## Data Availability

Not applicable.
